# Dynamic Targeting in Cancer Treatment

**DOI:** 10.3389/fphys.2019.00096

**Published:** 2019-02-14

**Authors:** Zhihui Wang, Thomas S. Deisboeck

**Affiliations:** ^1^Mathematics in Medicine Program, Houston Methodist Research Institute, Houston, TX, United States; ^2^Department of Imaging Physics, The University of Texas MD Anderson Cancer Center, Houston, TX, United States; ^3^Department of Radiology, Harvard-MIT (HST) Athinoula A. Martinos Center for Biomedical Imaging, Massachusetts General Hospital, Harvard Medical School, Charlestown, MA, United States

**Keywords:** drug discovery, mathematical modeling, network medicine, signaling pathway, therapeutic target, translational research

## Abstract

With the advent of personalized medicine, design and development of anti-cancer drugs that are specifically targeted to individual or sets of genes or proteins has been an active research area in both academia and industry. The underlying motivation for this approach is to interfere with several pathological crosstalk pathways in order to inhibit or at the very least control the proliferation of cancer cells. However, after initially conferring beneficial effects, if sub-lethal, these artificial perturbations in cell function pathways can inadvertently activate drug-induced up- and down-regulation of feedback loops, resulting in dynamic changes over time in the molecular network structure and potentially causing drug resistance as seen in clinics. Hence, the targets or their combined signatures should *also* change in accordance with the evolution of the network (reflected by changes to the structure and/or functional output of the network) over the course of treatment. This suggests the need for a “dynamic targeting” strategy aimed at optimizing tumor control by interfering with different molecular targets, at varying stages. Understanding the dynamic changes of this complex network under various perturbed conditions due to drug treatment is extremely challenging under experimental conditions let alone in clinical settings. However, mathematical modeling can facilitate studying these effects at the network level and beyond, and also accelerate comparison of the impact of different dosage regimens and therapeutic modalities prior to sizeable investment in risky and expensive clinical trials. A dynamic targeting strategy based on the use of mathematical modeling can be a new, exciting research avenue in the discovery and development of therapeutic drugs.

## Introduction

Cancer is a multifactorial and remarkably heterogeneous disease. Its initiation, progression, invasion, and metastasis processes all involve multiple molecular signaling mechanisms. The diversity of molecular and cellular properties across tumors from different patients, and even across cancer cells from the same patient, makes it extremely difficult to find a “one-size-fits-all” solution for therapeutic targeting of cancer. Thus, tailored targeted therapies based on each individual tumor’s characteristics are required in order to optimize treatment efficacy, minimize toxicity and drug side-effects, and ultimately lead to more cost-effective patient management by giving the most appropriate drugs at the optimum dose to every patient in need (Topol, 2014; Ryall and Tan, 2015). This is the essential concept of precision medicine.

From a systems biology perspective, cancer can be viewed as a network disease caused by dysregulation of molecular signaling pathways that determine various physiological cellular processes, such as growth, division, differentiation, and apoptosis (Creixell et al., 2012). These signaling pathways are not isolated from each other, but form a complex, interconnected network with numerous regulatory feedback loops and redundant pathways that together confer significant evolutionary robustness. Still, substantial advances have been made in development of targeted therapies based on detailed mechanistic understanding of these signaling networks, and as a result, some targeted drugs are emerging for clinical use (Yildirim et al., 2007; Hopkins, 2008). However, despite positive treatment responses in some patients, a large fraction of patients fail to benefit from these targeted therapies, even when molecular markers have been used to stratify patients into groups that are expected to respond to the therapy. Taking an approved ErbB-targeted drug (Herceptin) as an example, only about half of all patients with ErbB2-amplified metastatic breast cancer respond to the drug, and of those who do respond in the beginning, most eventually develop resistance (Garrett and Arteaga, 2011). This pattern of initial response followed by relapse is not unique to ErbB-targeted therapies, but has been seen for most molecularly targeted inhibitors (Al-Lazikani et al., 2012).

The disappointing response rate of targeted therapies is partly due to the resilience of oncogenic signaling networks that will often bypass a single hit through an abundance of the highly non-linear built-in feedback loops and alternative pathways that can compensate for therapeutic impact. To solve this “escape” problem, multiple therapies can be used together or in sequence, i.e., combination therapy, which can potentially block these parallel or alternative pathways activated in cancer cells (Fitzgerald et al., 2006). Since these therapeutic drugs may be administered at a smaller dosage for each individual drug, a combination therapy may stop oncogenic signaling or further delay resistance to treatments, while simultaneously minimizing overlapping toxicity. In theory, a combination approach would seem to have the potential to block alternative pathways, but, while there have been clinical successes, as with monotherapy they have not led to cure or long-term control for all patients (Chong and Janne, 2013; Yap et al., 2013; Sachs et al., 2016; Lopez and Banerji, 2017). One problem lies in the complexity of signaling networks, making it difficult to simply guess *a priori* which drug combinations are synergistically effective and which are not. Given the number of targeted drugs currently available and in clinical development, it is time-consuming and expensive to do unbiased screening of the large number of possible drug combinations at their clinically relevant dose and dosing schedules. Therefore, there is a major need for approaches that will allow us to identify effective drug combinations where two or more drugs work synergistically to suppress malfunctioning signaling.

Testing potentially clinically relevant drug combinations using mathematical models (see Box 1) offers a reasonable yet relatively simple and expeditious way to accomplish this task by computationally examining multiple targets through extensive parameter perturbation analyses (Araujo et al., 2005; Iyengar et al., 2012; Barbolosi et al., 2016). This approach allows for rapid and low-cost examination of the drug and target combination parameter space, including identification of potentially optimal drug combinations through mathematical methods, ultimately providing valuable insights which would be difficult (if not impossible) to achieve through traditional experimental and clinical trial methods and techniques. In the end, these models can help to narrow down and prioritize different target combinations prior to experimental validation.

**Box 1.**

Mathematical modeling of cancer treatment. Mathematical modeling is not only useful in providing mechanistic explanations of the observed data and generating valuable insights into how the molecular signaling network adapts under various perturbed conditions, it can also be used to derive new experimentally and clinically testable predictions. Data-driven modeling approaches that integrate statistical analysis of large-scale cancer multi-omics (e.g., genomics, proteomics, and other omics technologies) with clinical data have been used to identify key biological processes underlying cancer pathogenesis, prognostic biomarkers, and predictive signatures for drug response (Jerby and Ruppin, 2012; Casado et al., 2013; Niepel et al., 2013). On the other hand, mechanistic modeling approaches have been used to understand the roles of individual proteins in regulating cell fate and how signaling pathways interact to influence cancer progression (Prasasya et al., 2011; Hass et al., 2017), the dynamic interactions among cancer cells and between cells and the constantly changing microenvironment (Faratian et al., 2009; Klinger et al., 2013; Almendro et al., 2014; Leder et al., 2014), biophysical drug-cell interactions, and drug transport processes across tissues (Das et al., 2013; Pascal et al., 2013a,b; Koay et al., 2014; Frieboes et al., 2015; Wang et al., 2016; Brocato et al., 2018). In addition, mechanistic models are being generated to account for pharmacokinetics and pharmacodynamics to analyze drug action, dose-response relationships, and the time-course effect resulting from a drug dose, ultimately leading to the discovery of more effective dosing schedules (Swat et al., 2011; Vandamme et al., 2014; Wang et al., 2015a; Dogra et al., 2018). Furthermore, multiscale models of cancer have been developed to predict responses to treatments (perturbations), explain therapeutic resistance, and identify potential drug combinations across multiple biological scales, including at the molecular (such as gene regulatory and signal transduction networks), the cell, as well as at the tissue and whole organism scale (Wang and Deisboeck, 2008; Deisboeck et al., 2011; Wang et al., 2011a, 2015b; Gustafsson et al., 2014; Wolkenhauer et al., 2014; Wang and Maini, 2017). Overall, mathematical modeling paired with experimentation and clinical data analysis has led to substantial improvements in our understanding of the mechanistic basis for cancer progression and resistance development, advanced the systems-level interpretation of the pathophysiology relevant for drug discovery, and had an impact on the implementation and optimization of effective anticancer therapeutic strategies.

## Network Rewiring

It has been extensively reported that cancer cells or cell populations adapt or evolve in response to targeted therapies, in part by rewiring molecular mechanisms to overcome the inhibitory effects of initial treatments (Gillies et al., 2012; Logue and Morrison, 2012; Azad et al., 2015; Kolch et al., 2015; Stuhlmiller et al., 2015). This rewiring may involve alterations of signaling pathways, such as addition or deletion of edges in the network, modification of reaction rates, and changes in molecular concentrations, all of which may ultimately contribute to treatment resistance, either directly through rendering the drug ineffective or indirectly by leading to activation of alternative pro-survival or anti-apoptotic pathways. There are many other biological, biochemical, and biophysical factors [e.g., genetic alteration of individual cells, outgrowth of existing resistant subclones under selection pressure from treatment, altered effectors in DNA repair, pathway-independent acquired resistance, up-regulation of efflux pumps in cellular membranes, protein level oscillations within cells even in the absence of treatment, and physical barriers that may limit diffusive and convective drug transport (Minchinton and Tannock, 2006; Garraway and Janne, 2012; Brocato et al., 2014; Stewart et al., 2015; Cristini et al., 2017)] that may also contribute to cancer resistance to treatment, but rewiring of signaling pathways very likely plays an important role as a mechanism of acquired resistance. This implies that pharmacologically targeting the *compensatory* mechanisms (which have emerged due to this rewiring) should help to improve treatment efficacy and patient outcome (Solit and Rosen, 2011; Akhavan et al., 2013; Camidge et al., 2014).

Even before treatment, signaling networks are rewired in cancer cells compared to normal cells. Here, we briefly discuss several recent studies working toward understanding how signaling networks are rewired in cancer cells, and discuss how identification of these alterations can enable more effective cancer treatment. Creixell et al. (2015) performed systems-based research to evaluate whether cancer mutations perturb signaling networks and, if so, by what mechanisms. Using their collected global exome sequencing and proteomic data on the same set of cancer cell lines, some mutations were found to create new phosphorylation sites or destroy existing ones within a signaling network, or shift the network structure by upstream or downstream rewiring of the mutated signaling node. A variety of rewiring modes were identified, including constitutive activation and inactivation of kinase and SH2 domains, upstream and downstream rewiring of phosphorylation-based signaling, and the extinction and genesis of phosphorylation sites. Their results indicate that signaling networks are both dynamically and structurally rewired in cancer cells. More recently, Latysheva et al. (2016) investigated the interaction properties and structural features of more than two thousand fusion-forming proteins, and provided insight into the genome-scale molecular principles upon which fusion proteins could escape cell-death regulation and rewire signaling networks in cancer. Notably, using an integrated experimental and computational approach, Halasz et al. (2016) predicted and then validated feedback inhibition of insulin receptor substrate 1 (IRS1) by the kinase p70S6K in a zebrafish (*Danio rerio*) xenograft model to confer resistance to EGFR inhibition through extensive analysis of a perturbation data set targeting epidermal growth factor receptor (EGFR) and insulin-like growth factor 1 receptor (IGF1R) pathways in a panel of colorectal cancer cells. Some studies (Pandey et al., 2014) also point to transient or short-term pathway alterations resulting from one drug as causing increased sensitivity to a second drug delivered at a later time. Morton et al. (2014) designed a nanoparticle system that successfully delivered two different drugs with varying models of action to the tumor in a sequential manner. The first drug inhibited an oncogenic pathway through rewiring that sensitized the cells to DNA damage-induced apoptosis, and the second was a genotoxic drug that took advantage of the vulnerable state of the cancer cells to kill them with enhanced efficiency. Their results highlight how understanding the ways that signaling pathways change or rewire in response to treatment or drug exposure is essential for improving current translational and clinical research.

## Re-Identification and Re-Targeting

To predict cellular behavior, it is required to assess temporal- and state-based network dynamics in response to perturbations such as those induced by targeted drugs. It is thus highly rational to examine the newly rewired and altered molecular network [or networks, as some studies have found evidence that the dominant network is different at different tumor sites (Pestrin et al., 2009; Bhamidipati et al., 2013; Russo et al., 2017)], which arises after the first sub-lethal, targeted drug interventions, in order to identify and then reprioritize the targets. This will likely result in a new list of prioritized targets in the order of their importance in driving cancer cell survival and proliferation. The leading network modulator(s) on this new list should be prioritized as new drug targets in place of, or more likely in addition to, the previous top targets. In fact, rebiopsy at the time of progression of disease to guide changes in treatment has already been advocated in the literature (Yu et al., 2013; Planchard et al., 2015).

**FIGURE 1:**
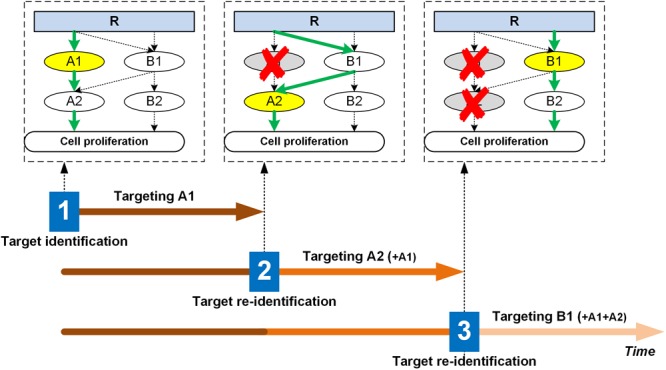
Illustration of the dynamic targeting strategy. The molecular signaling network changes or evolves with selective treatment. For instance, in this schematic, at time point 1, A1 emerges as the most critical node, hence during the first treatment period, A1 will be targeted with anti-A1. Assuming this to be of sub-lethal impact, the network rewires due to A1 inhibition, but the cell still finds a way to upregulate proliferation, so the treatment continues. At time point 2, A2 emerges as the top target, so the therapeutic regimen will attempt to inhibit A2 (together with A1) for the second treatment period. The network again rewires due to A2 inhibition, and the cell finds yet another way to bypass the A2 route and continues to proliferate. At time point 3, B1 becomes the top target, so the next treatment cycle will target B1 (together with A1 and A2). This process will continue until growth control is optimized and relapse to rapid replication does not occur. For each target at each treatment stage, exactly how much drug (dose) and how often to apply it (frequency) will require careful evaluation and should be different across patients. That is, other than depicted in the schematic for simplification purposes, the network adaptation is likely not hard-wired or rigidly dependent on external therapeutic pressure, but rather it undergoes a dynamic transition through an intrinsic optimization process. To manage side effects, a basic strategy could be to maximize the modulation effects on the top target specific to each treatment iteration, while keeping the “pressure” on prior targets at their respective “maintenance” minimum yet necessary dosing/frequency levels. Top targets are highlighted in yellow when the target identification process is performed. R: receptor; A1, A2, B1, B2: signaling molecules of the network.

This cascade of drug targeting, network rewiring, followed by subsequent target re-identification and reprioritization (potentially for multiple cycles), in our opinion, should be repeated during the entire course of treatment. Figure 1 shows a schematic of this process (to illustrate the concept, and not a specific treatment strategy much less a prediction), where for simplicity a single molecular intervention strategy is used at the beginning. While in reality the clinical situation in terms of signaling and rewiring will undoubtedly be much more complex, we however address two critical questions here. First, why not just take out the “important” molecules (e.g., A1, A2, and B1 in our schematic) at the onset of the therapeutic protocol to completely block the downstream signaling pathways that contribute to cell proliferation? The answer is two-fold – one, as discussed, we do not necessarily know *a priori* what “top” targets emerge as (conventional chemo- or radioactive, or advanced targeted) therapeutic interventions apply selective pressure on the cancer cells’ molecular network; secondly, this multi-target strategy will arguably be more toxic, and hence may cause more adverse side effects for the patient than necessary to achieve tumor control. Rather, the goal is to deliver optimal therapeutic efficacy at the minimum necessary level of side effects. As such, our dynamic targeting approach might just be the right answer in that it incrementally “probes” the network’s adaptive capabilities by applying a staggered amount of selective pressure. Also, effective targeting does not have to “take out” a target completely; it could instead be intended to modulate it up or down to redirect the network output. The second question is how frequently should the tumor system be re-examined in order to identify new targets or target combinations? While this is generally cancer type- and treatment-specific, it should also be patient-specific – yet remaining mindful of operational constraints and economics involved when translating this concept into a clinical setting. Still, in our opinion, every time a patient sees a diminishing therapeutic yield from, let alone fails a particular targeted treatment, the molecular network should be re-evaluated to potentially adjust the targeting strategy. We note that the timeline shown in Figure 1 is merely a schematic, and it follows that new network configurations (and thus the target hit-list) will differ in how fast they evolve, as would the drug dose and dosing schedules (determined uniquely for each drug delivered) for the individualized patient treatment plan.

In our dynamic approach, targets will emerge *sequentially* through “selection” imposed by targeted treatment and the perturbations it causes and reconfigurations the network stabilizes to. This is geared toward optimizing tumor growth control and as such differs from current combinatorics approaches (Gillies et al., 2012; Logue and Morrison, 2012), where the “most impactful” target combination is assessed once and then applied *a priori*, which should also incur more unexpected on-target or off-target side effects. We note that based on current reports on cell signaling (Tanay et al., 2005; Wei et al., 2016; Young et al., 2017), there are reasons to believe that there is some form of phase transition for network adaptability or maximum carrying capacity for the selection pressure or stress applied by a treatment, beyond which the cell simply dies. Rather than trying to kill all the cancer cells as efficaciously as possible, which is often impossible because of, e.g., detection limits and delivery challenges, our goal is to achieve maximum control over disease progression with minimal side effects, hence the sequential probing approach implemented in dynamic targeting.

Admittedly, there are many challenges in implementing this dynamic targeting strategy in current clinical practice. For example, immunotherapy is known to not always yield a tumor response within a time frame that other treatments may have shown, and some patients may experience initial increased size of tumor lesions with subsequent decreased tumor burden [this phenomenon is called pseudoprogression (Hodi et al., 2016)]. If a molecular targeted therapy is used together with immunotherapy, then we should give this type of combination treatment more time for re-evaluation of the patient; otherwise, it would prematurely eliminate treatments that might have been working but more slowly. As another quick example, if multiple clinical tests (genetic sequencing with high-throughput techniques, biopsy, imaging, etc.) are required for evaluating the tumor, then the question is whether it can be done in a reasonable time frame and at an acceptable risk for the patient, and if these additional assessments have a favorable cost to benefit ratio. Lastly, for any type of cancer, it should be kept in mind that only a subset of patients could benefit from a particular drug treatment. Hence, molecular diagnostics and imaging markers (Ransohoff and Gourlay, 2010; Reis-Filho and Pusztai, 2011; Jafari et al., 2017; Sepulveda et al., 2017) will be critical to correctly identify patient cohorts that are best suited for different targeted therapies, in addition to assessing response to therapy and monitoring patients for adverse drug reactions. Many other significant challenges related to further understanding tumor heterogeneity, tumor-host interactions, and immune response, etc. (Gatenby et al., 2010; Andre et al., 2013; Enriquez-Navas et al., 2016; Ibrahim-Hashim et al., 2017; Zhang et al., 2017) certainly exist in translating this strategy to clinical application. Further discussion of those challenges is beyond the scope of this article, as we only focus on introduction of a new concept, but it is worth emphasizing that many details with respect to technology, clinical care, regulation, and reimbursement need to be addressed in order to translate this concept into a reality.

To implement the dynamic targeting strategy, it would be prohibitive to evaluate the sheer number of mathematically possible drug target combinations multiple times over the course of treatment in preclinical animal models, let alone in a clinical setting. We therefore need, and should take full advantage of, large-scale unbiased methods based on mathematical modeling to evaluate and prioritize potential drug target combinations as early as possible. Indeed, mathematical network modeling has been helpful in identifying promising targets and effective combinations of existing targets (Wang et al., 2007, 2008, 2009, 2011b, 2012, 2014; Zhang et al., 2009; Miller et al., 2013; Wang and Deisboeck, 2014; Schoeberl et al., 2017). Once proven reliable, these models can be used to exhaustively test the efficacy of a large number of single drug and drug combinations by correlating signaling outputs with corresponding network perturbations in a dynamic fashion. Computer model simulations can be effectively integrated with quantitative wet lab studies to facilitate the process of identifying effective drug target combinations progressively over the course of treatment when treatment efficacy needs to be evaluated or a new treatment method is considered necessary; the mathematically narrowed down selection of individualized, computationally validated drug targets and combinations would then be handed over to conventional preclinical testing.

## Pilot Examples

We here discuss two recent examples to demonstrate the importance of dynamic targeting in cancer treatment. We note that both examples do not represent a full implementation of the dynamic targeting process. However, they reflect the necessity for novel approaches addressing network rewiring to find new, complementary drug targets or their combinations in an effort to truly improve survival and the probability of long-term remission if not cure in cancer treatment.

Lee et al. (2012) studied three cell lines from triple-negative breast cancer (i.e., estrogen receptor-, progesterone receptor-, and HER2 oncogene-negative) for their responses to seven genotoxic drugs and eight signaling inhibitors in various combinations and dosing schedules. They found that combination treatment with EGFR inhibitor (erlotinib) and DNA-damaging chemotherapy (doxorubicin) led to substantial killing of cancer cells, but only when the EGFR inhibition was used before the chemotherapy by at least 4 h. This combination treatment led to the rewiring of oncogenic signaling pathways, which has the potential to make cancer cells more susceptible to death. That is, the observed response relates to the dynamic effects on the molecular interaction network, which was rewired in response to EGFR inhibition, during which the cells once again became susceptible to death triggered by DNA damage. Since it was challenging to directly examine rewiring pathways by using wet lab experiments alone, they constructed a data-driven model based on partial least squares regression which was then used to correlate cellular responses with different forms of drug treatment. This study is significant, as it provides strong evidence that the timed application of signaling inhibitors causes the rewiring of signaling pathways in tumor cells and renders them more susceptible to subsequent chemotherapy. Other studies, such as Huether et al. (2005), also pointed to changes in apoptotic signaling pathways from a targeted therapy increasing chemotherapeutic sensitivity, with time dependence. Moreover, as also shown in other clinical research (Andre et al., 2003, 2004, 2009), this study by Lee et al. (2012) demonstrates that not only the selection of optimal drug combinations, but also the sequence and timing of the administration of the multiple therapeutic drugs were critical to maximize treatment efficacy. Goldman et al. (2015) also reported that if a chemotherapy drug pair is administered in the right temporal sequence combinations, the leading drug could induce a phenotypic cell state transition, thereby making the cancer vulnerable to the partner agent. Interestingly, they even proposed the use of mathematical modeling to optimize sequential treatment with two drugs to take advantage of rewiring in response to the first drug.

As another example, to understand the dynamic, non-linear behavior of signaling pathways in cancer, Bernardo-Faura et al. (2014) developed an adaptive model to study and predict changes in network architecture (i.e., topology) over time in response to drug treatment based on fuzzy logic, a method that has been widely used in computation and engineering. Using the model, they tested the dynamics of the mitogen-activated protein kinase (MAPK) pathway (which was composed of 10 signaling intermediates) against a dataset derived from a melanoma cell line that was exposed to different pharmacological kinase inhibitors over 4 days. They found that, although Sorafenib (an inhibitor) was considered to have the capability to prevent phosphorylation of MEK1/2, which should in turn suppress the activation of ERK1/2, the observed ERK1/2 profile was not consistently inhibited, suggesting a signaling rearrangement compared to the original MAPK pathway. While the rewired interaction could not be specifically identified with the model, the potential underlying biological mechanisms could range from genetic mechanisms (such as mutations) to spatiotemporal pathway regulations. This result also proved an interesting point: that some biological mechanisms may enable the cell to enhance certain pathways or prevent some reported interactions from happening in order to trigger a specific response, depending on the context or cell type (Jones et al., 2008). This adaptive modeling approach can be used to characterize dynamic signaling rearrangements that grant tumors the ability to maintain proliferation and develop resistance.

## Conclusion

Using the same drug or drug combinations throughout the course of treatment has been proven ineffective to overcome the pathway crosstalk and redundant signaling mechanisms, which are thought to be responsible (at least in part) for the modest responses observed in current trials of targeted therapies. Focusing on long-term tumor control rather than eradication, we introduce a *dynamic targeting strategy*, proposing that the target “signature” should change accordingly as the signaling network adapts during the course of treatment. Of course, this critically depends on being able to analyze the molecular networks readily and sufficiently, and mathematical models present an ideal platform for testing and optimizing drug combinations whenever target re-identification is needed. Ultimately, one may be able to predict the range of emerging target configurations, so that personalized, multi-tiered treatment can become proactive as opposed to being reactive to the network’s intrinsic ability to adapt. Compared to current preclinical and clinical oncology practice, our concept offers a faster, more effective, and thus arguably more economic approach to explore a large number of potential treatment strategies to identify an optimal, patient-specific therapeutic regimen.

## Author Contributions

Both authors made a substantial contribution to researching data for the article, discussions of content, writing, and reviewing and editing of the manuscript before submission.

## Conflict of Interest Statement

The authors declare that the research was conducted in the absence of any commercial or financial relationships that could be construed as a potential conflict of interest.

## References

[B1] Akhavan D. Pourzia A. L. Nourian A. A. Williams K. J. Nathanson D. Babic I. (2013). De-repression of PDGFRbeta transcription promotes acquired resistance to EGFR tyrosine kinase inhibitors in glioblastoma patients. *Cancer Discov.* 3 534–547. 10.1158/2159-8290.CD-12-0502 23533263 PMC3651754

[B2] Al-Lazikani B. Banerji U. Workman P. (2012). Combinatorial drug therapy for cancer in the post-genomic era. *Nat. Biotechnol.* 30 679–692. 10.1038/nbt.2284 22781697

[B3] Almendro V. Cheng Y. K. Randles A. Itzkovitz S. Marusyk A. Ametller E. (2014). Inference of tumor evolution during chemotherapy by computational modeling and in situ analysis of genetic and phenotypic cellular diversity. *Cell Rep.* 6 514–527. 10.1016/j.celrep.2013.12.041 24462293 PMC3928845

[B4] Andre F. Dieci M. V. Dubsky P. Sotiriou C. Curigliano G. Denkert C. (2013). Molecular pathways: involvement of immune pathways in the therapeutic response and outcome in breast cancer. *Clin. Cancer Res.* 19 28–33. 10.1158/1078-0432.CCR-11-2701 23258741

[B5] Andre T. Boni C. Mounedji-Boudiaf L. Navarro M. Tabernero J. Hickish T. (2004). Oxaliplatin, fluorouracil, and leucovorin as adjuvant treatment for colon cancer. *N. Engl. J. Med.* 350 2343–2351. 10.1056/NEJMoa032709 15175436

[B6] Andre T. Boni C. Navarro M. Tabernero J. Hickish T. Topham C. (2009). Improved overall survival with oxaliplatin, fluorouracil, and leucovorin as adjuvant treatment in stage II or III colon cancer in the MOSAIC trial. *J. Clin. Oncol.* 27 3109–3116. 10.1200/JCO.2008.20.6771 19451431

[B7] Andre T. Colin P. Louvet C. Gamelin E. Bouche O. Achille E. (2003). Semimonthly versus monthly regimen of fluorouracil and leucovorin administered for 24 or 36 weeks as adjuvant therapy in stage II and III colon cancer: results of a randomized trial. *J. Clin. Oncol.* 21 2896–2903. 10.1200/JCO.2003.10.065 12885807

[B8] Araujo R. P. Petricoin E. F. Liotta L. A. (2005). A mathematical model of combination therapy using the EGFR signaling network. *Biosystems* 80 57–69. 10.1016/j.biosystems.2004.10.002 15740835

[B9] Azad A. K. Lawen A. Keith J. M. (2015). Prediction of signaling cross-talks contributing to acquired drug resistance in breast cancer cells by bayesian statistical modeling. *BMC Syst. Biol.* 9:2. 10.1186/s12918-014-0135-x 25599599 PMC4307189

[B10] Barbolosi D. Ciccolini J. Lacarelle B. Barlesi F. Andre N. (2016). Computational oncology–mathematical modelling of drug regimens for precision medicine. *Nat. Rev. Clin. Oncol.* 13 242–254. 10.1038/nrclinonc.2015.204 26598946

[B11] Bernardo-Faura M. Massen S. Falk C. S. Brady N. R. Eils R. (2014). Data-derived modeling characterizes plasticity of MAPK signaling in melanoma. *PLoS Comput. Biol.* 10:e1003795. 10.1371/journal.pcbi.1003795 25188314 PMC4154640

[B12] Bhamidipati P. K. Kantarjian H. Cortes J. Cornelison A. M. Jabbour E. (2013). Management of imatinib-resistant patients with chronic myeloid leukemia. *Ther. Adv. Hematol.* 4 103–117. 10.1177/2040620712468289 23610618 PMC3629755

[B13] Brocato T. Dogra P. Koay E. J. Day A. Chuang Y. L. Wang Z. (2014). Understanding drug resistance in breast cancer with mathematical oncology. *Curr. Breast Cancer Rep.* 6 110–120. 10.1007/s12609-014-0143-2 24891927 PMC4039558

[B14] Brocato T. A. Coker E. N. Durfee P. N. Lin Y. S. Townson J. Wyckoff E. F. (2018). Understanding the connection between nanoparticle uptake and cancer treatment efficacy using mathematical modeling. *Sci. Rep.* 8:7538. 10.1038/s41598-018-25878-8 29795392 PMC5967303

[B15] Camidge D. R. Pao W. Sequist L. V. (2014). Acquired resistance to TKIs in solid tumours: learning from lung cancer. *Nat. Rev. Clin. Oncol.* 11 473–481. 10.1038/nrclinonc.2014.104 24981256

[B16] Casado P. Rodriguez-Prados J. C. Cosulich S. C. Guichard S. Vanhaesebroeck B. Joel S. (2013). Kinase-substrate enrichment analysis provides insights into the heterogeneity of signaling pathway activation in leukemia cells. *Sci. Signal.* 6 rs6. 10.1126/scisignal.2003573 23532336

[B17] Chong C. R. Janne P. A. (2013). The quest to overcome resistance to EGFR-targeted therapies in cancer. *Nat. Med.* 19 1389–1400. 10.1038/nm.3388 24202392 PMC4049336

[B18] Creixell P. Schoof E. M. Erler J. T. Linding R. (2012). Navigating cancer network attractors for tumor-specific therapy. *Nat. Biotechnol.* 30 842–848. 10.1038/nbt.2345 22965061

[B19] Creixell P. Schoof E. M. Simpson C. D. Longden J. Miller C. J. Lou H. J. (2015). Kinome-wide decoding of network-attacking mutations rewiring cancer signaling. *Cell* 163 202–217. 10.1016/j.cell.2015.08.056 26388441 PMC4644236

[B20] Cristini V. Koay E. Wang Z. (2017). *An Introduction to Physical Oncology: How Mechanistic Mathematical Modeling Can Improve Cancer Therapy Outcomes*. Boca Raton, FL: CRC Press 10.4324/9781315374499

[B21] Das H. Wang Z. Niazi M. K. Aggarwal R. Lu J. Kanji S. (2013). Impact of diffusion barriers to small cytotoxic molecules on the efficacy of immunotherapy in breast cancer. *PLoS One* 8:e61398. 10.1371/journal.pone.0061398 23620747 PMC3631240

[B22] Deisboeck T. S. Wang Z. Macklin P. Cristini V. (2011). Multiscale cancer modeling. *Annu. Rev. Biomed Eng.* 13 127–155. 10.1146/annurev-bioeng-071910-124729 21529163 PMC3883359

[B23] Dogra P. Adolphi N. L. Wang Z. Lin Y. S. Butler K. S. Durfee P. N. (2018). Establishing the effects of mesoporous silica nanoparticle properties on in vivo disposition using imaging-based pharmacokinetics. *Nat. Commun.* 9:4551. 10.1038/s41467-018-06730-z 30382084 PMC6208419

[B24] Enriquez-Navas P. M. Kam Y. Das T. Hassan S. Silva A. Foroutan P. (2016). Exploiting evolutionary principles to prolong tumor control in preclinical models of breast cancer. *Sci. Transl. Med.* 8:327ra24. 10.1126/scitranslmed.aad7842 26912903 PMC4962860

[B25] Faratian D. Goltsov A. Lebedeva G. Sorokin A. Moodie S. Mullen P. (2009). Systems biology reveals new strategies for personalizing cancer medicine and confirms the role of PTEN in resistance to trastuzumab. *Cancer Res.* 69 6713–6720. 10.1158/0008-5472.CAN-09-0777 19638581

[B26] Fitzgerald J. B. Schoeberl B. Nielsen U. B. Sorger P. K. (2006). Systems biology and combination therapy in the quest for clinical efficacy. *Nat. Chem. Biol.* 2 458–466. 10.1038/nchembio817 16921358

[B27] Frieboes H. B. Smith B. R. Wang Z. Kotsuma M. Ito K. Day A. (2015). Predictive modeling of drug response in non-hodgkin’s lymphoma. *PLoS One* 10:e0129433. 10.1371/journal.pone.0129433 26061425 PMC4464754

[B28] Garraway L. A. Janne P. A. (2012). Circumventing cancer drug resistance in the era of personalized medicine. *Cancer Discov.* 2 214–226. 10.1158/2159-8290.CD-12-0012 22585993

[B29] Garrett J. T. Arteaga C. L. (2011). Resistance to HER2-directed antibodies and tyrosine kinase inhibitors: mechanisms and clinical implications. *Cancer Biol. Ther.* 11 793–800. 10.4161/cbt.11.9.15045 21307659 PMC3230295

[B30] Gatenby R. A. Gillies R. J. Brown J. S. (2010). Evolutionary dynamics of cancer prevention. *Nat. Rev. Cancer* 10 526–527. 10.1038/nrc2892 21137109 PMC3744108

[B31] Gillies R. J. Verduzco D. Gatenby R. A. (2012). Evolutionary dynamics of carcinogenesis and why targeted therapy does not work. *Nat. Rev. Cancer* 12 487–493. 10.1038/nrc3298 22695393 PMC4122506

[B32] Goldman A. Majumder B. Dhawan A. Ravi S. Goldman D. Kohandel M. (2015). Temporally sequenced anticancer drugs overcome adaptive resistance by targeting a vulnerable chemotherapy-induced phenotypic transition. *Nat. Commun.* 6:6139. 10.1038/ncomms7139 25669750 PMC4339891

[B33] Gustafsson M. Nestor C. E. Zhang H. Barabasi A. L. Baranzini S. Brunak S. (2014). Modules, networks and systems medicine for understanding disease and aiding diagnosis. *Genome Med.* 6:82. 10.1186/s13073-014-0082-6 25473422 PMC4254417

[B34] Halasz M. Kholodenko B. N. Kolch W. Santra T. (2016). Integrating network reconstruction with mechanistic modeling to predict cancer therapies. *Sci. Signal.* 9:ra114. 27879396 10.1126/scisignal.aae0535

[B35] Hass H. Masson K. Wohlgemuth S. Paragas V. Allen J. E. Sevecka M. (2017). Predicting ligand-dependent tumors from multi-dimensional signaling features. *NPJ Syst. Biol. Appl.* 3:27. 10.1038/s41540-017-0030-3 28944080 PMC5607260

[B36] Hodi F. S. Hwu W. J. Kefford R. Weber J. S. Daud A. Hamid O. (2016). Evaluation of immune-related response criteria and RECIST v1.1 in patients with advanced melanoma treated with pembrolizumab. *J. Clin. Oncol.* 34 1510–1517. 10.1200/JCO.2015.64.0391 26951310 PMC5070547

[B37] Hopkins A. L. (2008). Network pharmacology: the next paradigm in drug discovery. *Nat. Chem. Biol.* 4 682–690. 10.1038/nchembio.118 18936753

[B38] Huether A. Hopfner M. Sutter A. P. Schuppan D. Scherubl H. (2005). Erlotinib induces cell cycle arrest and apoptosis in hepatocellular cancer cells and enhances chemosensitivity towards cytostatics. *J. Hepatol.* 43 661–669. 10.1016/j.jhep.2005.02.040 16023762

[B39] Ibrahim-Hashim A. Robertson-Tessi M. Enriquez-Navas P. M. Damaghi M. Balagurunathan Y. Wojtkowiak J. W. (2017). Defining cancer subpopulations by adaptive strategies rather than molecular properties provides novel insights into intratumoral evolution. *Cancer Res.* 77 2242–2254. 10.1158/0008-5472.CAN-16-2844 28249898 PMC6005351

[B40] Iyengar R. Zhao S. Chung S. W. Mager D. E. Gallo J. M. (2012). Merging systems biology with pharmacodynamics. *Sci. Transl. Med.* 4:126ps7. 10.1126/scitranslmed.3003563 22440734 PMC3405973

[B41] Jafari S. H. Saadatpour Z. Salmaninejad A. Momeni F. Mokhtari M. Nahand J. S. (2017). Breast cancer diagnosis: imaging techniques and biochemical markers. *J. Cell Physiol.* 233 5200–5213. 10.1002/jcp.26379 29219189

[B42] Jerby L. Ruppin E. (2012). Predicting drug targets and biomarkers of cancer via genome-scale metabolic modeling. *Clin. Cancer Res.* 18 5572–5584. 10.1158/1078-0432.CCR-12-1856 23071359

[B43] Jones S. Zhang X. Parsons D. W. Lin J. C. Leary R. J. Angenendt P. (2008). Core signaling pathways in human pancreatic cancers revealed by global genomic analyses. *Science* 321 1801–1806. 10.1126/science.1164368 18772397 PMC2848990

[B44] Klinger B. Sieber A. Fritsche-Guenther R. Witzel F. Berry L. Schumacher D. (2013). Network quantification of EGFR signaling unveils potential for targeted combination therapy. *Mol. Syst. Biol.* 9:673. 10.1038/msb.2013.29 23752269 PMC3964313

[B45] Koay E. J. Truty M. J. Cristini V. Thomas R. M. Chen R. Chatterjee D. (2014). Transport properties of pancreatic cancer describe gemcitabine delivery and response. *J. Clin. Invest.* 124 1525–1536. 10.1172/JCI73455 24614108 PMC3973100

[B46] Kolch W. Halasz M. Granovskaya M. Kholodenko B. N. (2015). The dynamic control of signal transduction networks in cancer cells. *Nat. Rev. Cancer* 15 515–527. 10.1038/nrc3983 26289315

[B47] Latysheva N. S. Oates M. E. Maddox L. Flock T. Gough J. Buljan M. (2016). Molecular principles of gene fusion mediated rewiring of protein interaction networks in cancer. *Mol. Cell* 63 579–592. 10.1016/j.molcel.2016.07.008 27540857 PMC5003813

[B48] Leder K. Pitter K. Laplant Q. Hambardzumyan D. Ross B. D. Chan T. A. (2014). Mathematical modeling of PDGF-driven glioblastoma reveals optimized radiation dosing schedules. *Cell* 156 603–616. 10.1016/j.cell.2013.12.029 24485463 PMC3923371

[B49] Lee M. J. Ye A. S. Gardino A. K. Heijink A. M. Sorger P. K. Macbeath G. (2012). Sequential application of anticancer drugs enhances cell death by rewiring apoptotic signaling networks. *Cell* 149 780–794. 10.1016/j.cell.2012.03.031 22579283 PMC3501264

[B50] Logue J. S. Morrison D. K. (2012). Complexity in the signaling network: insights from the use of targeted inhibitors in cancer therapy. *Genes Dev.* 26 641–650. 10.1101/gad.186965.112 22474259 PMC3323875

[B51] Lopez J. S. Banerji U. (2017). Combine and conquer: challenges for targeted therapy combinations in early phase trials. *Nat. Rev. Clin. Oncol.* 14 57–66. 10.1038/nrclinonc.2016.96 27377132 PMC6135233

[B52] Miller M. L. Molinelli E. J. Nair J. S. Sheikh T. Samy R. Jing X. (2013). Drug synergy screen and network modeling in dedifferentiated liposarcoma identifies CDK4 and IGF1R as synergistic drug targets. *Sci. Signal.* 6:ra85. 10.1126/scisignal.2004014 24065146 PMC4000046

[B53] Minchinton A. I. Tannock I. F. (2006). Drug penetration in solid tumours. *Nat. Rev. Cancer* 6 583–592. 10.1038/nrc1893 16862189

[B54] Morton S. W. Lee M. J. Deng Z. J. Dreaden E. C. Siouve E. Shopsowitz K. E. (2014). A nanoparticle-based combination chemotherapy delivery system for enhanced tumor killing by dynamic rewiring of signaling pathways. *Sci. Signal.* 7:ra44. 10.1126/scisignal.2005261 24825919 PMC4138219

[B55] Niepel M. Hafner M. Pace E. A. Chung M. Chai D. H. Zhou L. (2013). Profiles of Basal and stimulated receptor signaling networks predict drug response in breast cancer lines. *Sci. Signal.* 6:ra84. 10.1126/scisignal.2004379 24065145 PMC3845839

[B56] Pandey A. Kulkarni A. Roy B. Goldman A. Sarangi S. Sengupta P. (2014). Sequential application of a cytotoxic nanoparticle and a PI3K inhibitor enhances antitumor efficacy. *Cancer Res.* 74 675–685. 10.1158/0008-5472.CAN-12-3783 24121494 PMC3946433

[B57] Pascal J. Ashley C. E. Wang Z. Brocato T. A. Butner J. D. Carnes E. C. (2013a). Mechanistic modeling identifies drug-uptake history as predictor of tumor drug resistance and nano-carrier-mediated response. *ACS Nano* 7 11174–11182. 10.1021/nn4048974 24187963 PMC3891887

[B58] Pascal J. Bearer E. L. Wang Z. Koay E. J. Curley S. A. Cristini V. (2013b). Mechanistic patient-specific predictive correlation of tumor drug response with microenvironment and perfusion measurements. *Proc. Natl. Acad. Sci. U.S.A.* 110 14266–14271. 10.1073/pnas.1300619110 23940372 PMC3761643

[B59] Pestrin M. Bessi S. Galardi F. Truglia M. Biggeri A. Biagioni C. (2009). Correlation of HER2 status between primary tumors and corresponding circulating tumor cells in advanced breast cancer patients. *Breast Cancer Res. Treat.* 118 523–530. 10.1007/s10549-009-0461-7 19597704

[B60] Planchard D. Loriot Y. Andre F. Gobert A. Auger N. Lacroix L. (2015). EGFR-independent mechanisms of acquired resistance to AZD9291 in EGFR T790M-positive NSCLC patients. *Ann. Oncol.* 26 2073–2078. 10.1093/annonc/mdv319 26269204

[B61] Prasasya R. D. Tian D. Kreeger P. K. (2011). Analysis of cancer signaling networks by systems biology to develop therapies. *Semin. Cancer Biol.* 21 200–206. 10.1016/j.semcancer.2011.04.001 21511035

[B62] Ransohoff D. F. Gourlay M. L. (2010). Sources of bias in specimens for research about molecular markers for cancer. *J. Clin. Oncol.* 28 698–704. 10.1200/JCO.2009.25.6065 20038718 PMC2816003

[B63] Reis-Filho J. S. Pusztai L. (2011). Gene expression profiling in breast cancer: classification, prognostication, and prediction. *Lancet* 378 1812–1823. 10.1016/S0140-6736(11)61539-0 22098854

[B64] Russo A. Franchina T. Ricciardi G. R. R. Smiroldo V. Picciotto M. Zanghi M. (2017). Third generation EGFR TKIs in EGFR-mutated NSCLC: where are we now and where are we going. *Crit. Rev. Oncol. Hematol.* 117 38–47. 10.1016/j.critrevonc.2017.07.003 28807234

[B65] Ryall K. A. Tan A. C. (2015). Systems biology approaches for advancing the discovery of effective drug combinations. *J. Cheminform.* 7:7. 10.1186/s13321-015-0055-9 25741385 PMC4348553

[B66] Sachs J. R. Mayawala K. Gadamsetty S. Kang S. P. De Alwis D. P. (2016). Optimal dosing for targeted therapies in oncology: drug development cases leading by example. *Clin. Cancer Res.* 22 1318–1324. 10.1158/1078-0432.CCR-15-1295 26597302

[B67] Schoeberl B. Kudla A. Masson K. Kalra A. Curley M. Finn G. (2017). Systems biology driving drug development: from design to the clinical testing of the anti-ErbB3 antibody seribantumab (MM-121). *NPJ Syst. Biol. Appl.* 3:16034. 10.1038/npjsba.2016.34 28725482 PMC5516865

[B68] Sepulveda A. R. Hamilton S. R. Allegra C. J. Grody W. Cushman-Vokoun A. M. Funkhouser W. K. (2017). Molecular biomarkers for the evaluation of colorectal cancer: guideline from the american society for clinical pathology, college of american pathologists, association for molecular pathology, and the american society of clinical oncology. *J. Clin. Oncol.* 35 1453–1486. 10.1200/JCO.2016.71.9807 28165299

[B69] Solit D. B. Rosen N. (2011). Resistance to BRAF inhibition in melanomas. *N. Engl. J. Med.* 364 772–774. 10.1056/NEJMcibr1013704 21345109

[B70] Stewart E. L. Mascaux C. Pham N. A. Sakashita S. Sykes J. Kim L. (2015). Clinical utility of patient-derived xenografts to determine biomarkers of prognosis and map resistance pathways in EGFR-mutant lung adenocarcinoma. *J. Clin. Oncol.* 33 2472–2480. 10.1200/JCO.2014.60.1492 26124487

[B71] Stuhlmiller T. J. Miller S. M. Zawistowski J. S. Nakamura K. Beltran A. S. Duncan J. S. (2015). Inhibition of lapatinib-induced kinome reprogramming in ERBB2-positive breast cancer by targeting BET family bromodomains. *Cell Rep.* 11 390–404. 10.1016/j.celrep.2015.03.037 25865888 PMC4408261

[B72] Swat M. Kielbasa S. M. Polak S. Olivier B. Bruggeman F. J. Tulloch M. Q. (2011). What it takes to understand and cure a living system: computational systems biology and a systems biology-driven pharmacokinetics-pharmacodynamics platform. *Interface Focus* 1 16–23. 10.1098/rsfs.2010.0011 22419971 PMC3262244

[B73] Tanay A. Regev A. Shamir R. (2005). Conservation and evolvability in regulatory networks: the evolution of ribosomal regulation in yeast. *Proc. Natl. Acad. Sci. U.S.A.* 102 7203–7208. 10.1073/pnas.0502521102 15883364 PMC1091753

[B74] Topol E. J. (2014). Individualized medicine from prewomb to tomb. *Cell* 157 241–253. 10.1016/j.cell.2014.02.012 24679539 PMC3995127

[B75] Vandamme D. Minke B. A. Fitzmaurice W. Kholodenko B. N. Kolch W. (2014). Systems biology-embedded target validation: improving efficacy in drug discovery. *Wiley Interdiscip Rev. Syst. Biol. Med.* 6 1–11. 10.1002/wsbm.1253 24214316

[B76] Wang Z. Birch C. M. Deisboeck T. S. (2008). Cross-scale sensitivity analysis of a non-small cell lung cancer model: linking molecular signaling properties to cellular behavior. *Biosystems* 92 249–258. 10.1016/j.biosystems.2008.03.002 18448237 PMC2430419

[B77] Wang Z. Birch C. M. Sagotsky J. Deisboeck T. S. (2009). Cross-scale, cross-pathway evaluation using an agent-based non-small cell lung cancer model. *Bioinformatics* 25 2389–2396. 10.1093/bioinformatics/btp416 19578172 PMC2735669

[B78] Wang Z. Bordas V. Deisboeck T. S. (2011a). Discovering molecular targets in cancer with multiscale modeling. *Drug Dev. Res.* 72 45–52. 21572568 10.1002/ddr.20401 PMC3092304

[B79] Wang Z. Bordas V. Deisboeck T. S. (2011b). Identification of critical molecular components in a multiscale cancer model based on the integration of monte carlo. Resampling, and ANOVA. *Front. Physiol.* 2:35. 10.3389/fphys.2011.00035 21779251 PMC3132643

[B80] Wang Z. Bordas V. Sagotsky J. Deisboeck T. S. (2012). Identifying therapeutic targets in a combined EGFR-TGFbetaR signalling cascade using a multiscale agent-based cancer model. *Math. Med. Biol.* 29 95–108. 10.1093/imammb/dqq023 21147846 PMC3499073

[B81] Wang Z. Butner J. D. Cristini V. Deisboeck T. S. (2015a). Integrated PK-PD and agent-based modeling in oncology. *J. Pharmacokinet. Pharmacodyn.* 42 179–189. 10.1007/s10928-015-9403-7 25588379 PMC4985529

[B82] Wang Z. Butner J. D. Kerketta R. Cristini V. Deisboeck T. S. (2015b). Simulating cancer growth with multiscale agent-based modeling. *Semin. Cancer Biol.* 30 70–78. 10.1016/j.semcancer.2014.04.001 24793698 PMC4216775

[B83] Wang Z. Deisboeck T. S. (2008). Computational modeling of brain tumors: discrete, continuum or hybrid? *Sci. Model. Simul.* 15 381–393. 10.1007/s10820-008-9094-0

[B84] Wang Z. Deisboeck T. S. (2014). Mathematical modeling in cancer drug discovery. *Drug Discov. Today* 19 145–150. 10.1016/j.drudis.2013.06.015 23831857

[B85] Wang Z. Deisboeck T. S. Cristini V. (2014). Development of a sampling-based global sensitivity analysis workflow for multiscale computational cancer models. *IET Syst. Biol.* 8 191–197. 10.1049/iet-syb.2013.0026 25257020 PMC4180114

[B86] Wang Z. Kerketta R. Chuang Y. L. Dogra P. Butner J. D. Brocato T. A. (2016). Theory and experimental validation of a spatio-temporal model of chemotherapy transport to enhance tumor cell kill. *PLoS Comput. Biol.* 12:e1004969. 10.1371/journal.pcbi.1004969 27286441 PMC4902302

[B87] Wang Z. Maini P. K. (2017). Editorial: special section on multiscale cancer modeling. *IEEE Trans. Biomed Eng.* 64 501–503. 10.1109/TBME.2017.2655439 28824199 PMC5557380

[B88] Wang Z. Zhang L. Sagotsky J. Deisboeck T. S. (2007). Simulating non-small cell lung cancer with a multiscale agent-based model. *Theor. Biol. Med. Model.* 4:50. 10.1186/1742-4682-4-50 18154660 PMC2259313

[B89] Wei W. Shin Y. S. Xue M. Matsutani T. Masui K. Yang H. (2016). Single-cell phosphoproteomics resolves adaptive signaling dynamics and informs targeted combination therapy in glioblastoma. *Cancer Cell* 29 563–573. 10.1016/j.ccell.2016.03.012 27070703 PMC4831071

[B90] Wolkenhauer O. Auffray C. Brass O. Clairambault J. Deutsch A. Drasdo D. (2014). Enabling multiscale modeling in systems medicine. *Genome Med.* 6:21. 10.1186/gm538 25031615 PMC4062045

[B91] Yap T. A. Omlin A. De Bono J. S. (2013). Development of therapeutic combinations targeting major cancer signaling pathways. *J. Clin. Oncol.* 31 1592–1605. 10.1200/JCO.2011.37.6418 23509311

[B92] Yildirim M. A. Goh K. I. Cusick M. E. Barabasi A. L. Vidal M. (2007). Drug-target network. *Nat. Biotechnol.* 25 1119–1126. 10.1038/nbt1338 17921997

[B93] Young H. L. Rowling E. J. Bugatti M. Giurisato E. Luheshi N. Arozarena I. (2017). An adaptive signaling network in melanoma inflammatory niches confers tolerance to MAPK signaling inhibition. *J. Exp. Med.* 214 1691–1710. 10.1084/jem.20160855 28450382 PMC5460994

[B94] Yu H. A. Arcila M. E. Rekhtman N. Sima C. S. Zakowski M. F. Pao W. (2013). Analysis of tumor specimens at the time of acquired resistance to EGFR-TKI therapy in 155 patients with EGFR-mutant lung cancers. *Clin. Cancer Res.* 19 2240–2247. 10.1158/1078-0432.CCR-12-2246 23470965 PMC3630270

[B95] Zhang J. Cunningham J. J. Brown J. S. Gatenby R. A. (2017). Integrating evolutionary dynamics into treatment of metastatic castrate-resistant prostate cancer. *Nat. Commun.* 8:1816. 10.1038/s41467-017-01968-5 29180633 PMC5703947

[B96] Zhang L. Strouthos C. G. Wang Z. Deisboeck T. S. (2009). Simulating brain tumor heterogeneity with a multiscale agent-based model: linking molecular signatures, phenotypes and expansion rate. *Math. Comput. Model.* 49 307–319. 10.1016/j.mcm.2008.05.011 20047002 PMC2653254

